# What Do Older People Do When Sitting and Why? Implications for Decreasing Sedentary Behavior

**DOI:** 10.1093/geront/gny020

**Published:** 2018-05-15

**Authors:** Victoria J Palmer, Cindy M Gray, Claire F Fitzsimons, Nanette Mutrie, Sally Wyke, Ian J Deary, Geoff Der, Sebastien F M Chastin, Dawn A Skelton, Seniors USP Team, Dawn A Skelton, Sebastien Chastin, Simon Cox, Elaine Coulter, Iva Čukić, Philippa Dall, Ian Deary, Geoff Der, Manon Dontje, Claire Fitzsimons, Catharine Gale, Jason Gill, Malcolm Granat, Cindy Gray, Carolyn Greig, Elaine Hindle, Karen Laird, Gillian Mead, Nanette Mutrie, Victoria Palmer, Ratko Radakovic, Naveed Sattar, Richard Shaw, John Starr, Sally Stewart, Sally Wyke

**Affiliations:** 1 Institute of Health and Wellbeing, College of Social Sciences, University of Glasgow, UK; 2 Physical Activity for Health Research Centre, Institute Sport, Physical Education and Health Sciences; 3 Centre for Cognitive Ageing and Cognitive Epidemiology, Department of Psychology, University of Edinburgh, UK; 4 MRC/CSO Social and Public Health Sciences Unit, University of Glasgow, UK; 5 Institute for Applied Health Research, School of Health and Life Sciences, Glasgow Caledonian University, UK; 6 Department of Sport and Movement Sciences, Ghent University, Belgium

**Keywords:** Qualitative, Social practice model, Ecological model, Intervention, Experiences

## Abstract

**Background and Objectives:**

Sitting less can reduce older adults’ risk of ill health and disability. Effective sedentary behavior interventions require greater understanding of what older adults do when sitting (and not sitting), and why. This study compares the types, context, and role of sitting activities in the daily lives of older men and women who sit more or less than average.

**Research Design and Methods:**

Semistructured interviews with 44 older men and women of different ages, socioeconomic status, and objectively measured sedentary behavior were analyzed using social practice theory to explore the multifactorial, inter-relational influences on their sedentary behavior. Thematic frameworks facilitated between-group comparisons.

**Results:**

Older adults described many different leisure time, household, transport, and occupational sitting and non-sitting activities. Leisure-time sitting in the home (e.g., watching TV) was most common, but many non-sitting activities, including “pottering” doing household chores, also took place at home. Other people and access to leisure facilities were associated with lower sedentary behavior. The distinction between being busy/not busy was more important to most participants than sitting/not sitting, and informed their judgments about high-value “purposeful” (social, cognitively active, restorative) sitting and low-value “passive” sitting. Declining physical function contributed to temporal sitting patterns that did not vary much from day-to-day.

**Discussion and Implications:**

Sitting is associated with cognitive, social, and/or restorative benefits, embedded within older adults’ daily routines, and therefore difficult to change. Useful strategies include supporting older adults to engage with other people and local facilities outside the home, and break up periods of passive sitting at home.

Sedentary behavior is defined as any waking activity in a sitting or reclining posture where energy expenditure is ≤1.5 metabolic equivalents (Sedentary Behaviour Research Network, 2012). Prolonged sedentary behavior throughout the day increases risk of poor health, even in people who are physically active (Gennuso, Gangnon, Matthews, Thraen-Borowski, & Colbert, 2013). Older adults are one of the most sedentary age groups, spending more than 60% (8.5–9.6 hr) of their waking day sitting (Harvey, Chastin, & Skelton, 2013, 2015), placing them at increased risk of all-cause mortality, metabolic syndrome, and obesity (Rezende, Rey-López, Matsudo, & do Carmo Luiz, 2014;Wullems, Verschueren, Degens, Morse, & Onambele, 2016). Reducing or breaking up periods of prolonged sitting therefore has potential to improve older adults’ health, and UK physical activity guidance now advises older adults to limit the amount of time they spend sedentary (Department of Health, 2011). However, there is little evidence on which to guide sedentary behavior interventions (Martin et al., 2015).

Previous research has provided some insight into the type and context of sedentary behavior and has shown that sitting activities older adults typically engage in include watching TV, reading, eating meals, using the computer and transport (Lenz, 2014). One study using time-lapse cameras suggested that older adults often sit most in the afternoon and evening (compared with the morning), and when they are alone at home (Leask, Harvey, Skelton, & Chastin, 2015). However, to develop interventions to reduce their sedentary behavior, we also need to know*why* older adults spend time sitting and not sitting and how they understand sedentary and nonsedentary behavior.

A small number of qualitative studies have begun to explore factors that influence older adults’ sedentary behavior. These studies suggest that older adults enjoy, and recognize the physical, social, and mental benefits, of some sitting activities (e.g., doing arts, crafts and puzzles, and going to the theatre;Chastin, Fitzpatrick, Andrews, & DiCroce, 2014;McEwan, Tam-Seto, & Dogra, 2016), but view excessive sitting as unhealthy. Many older adults report how aches and pains, poor health, and lack of energy make them sit more (Chastin et al., 2014;Dogra, Tam-Seto, & Weir, 2016;Greenwood-Hickman, Renz, & Rosenberg, 2015;McEwan et al., 2016), and some report using sitting to manage health conditions and energy expenditure (Chastin et al., 2014;Leask, Sandlund, Skelton, Tulle, & Chastin, 2016).

Societal attitudes toward aging can result in older adults feeling pressure to sit (Chastin et al., 2014;Greenwood-Hickman et al., 2015;McEwan et al., 2016). For example, lack of knowledge about the benefits of sitting less, mean that family, friends, and carers often encourage them to sit more (Chastin et al., 2014;Greenwood-Hickman et al., 2015), and many community activities aimed at older adults are sitting based (Chastin et al., 2014). Older adults blame bad weather and lack of outdoor seating for limiting their ability to go for a walk (Chastin et al., 2014;Greenwood-Hickman et al., 2015;McEwan et al., 2016), and that poor public transport and lack of availability of/information about community-based resources lead them to sit more (Chastin et al., 2014;Dogra et al., 2016). However, existing studies have mainly included women (Chastin et al., 2014;Dogra et al., 2016;Greenwood-Hickman et al., 2015;Leask et al., 2016;McEwan et al., 2016), people who were well-educated or from more affluent backgrounds (Chastin et al., 2014;Dogra et al., 2016;Greenwood-Hickman et al., 2015;McEwan et al., 2016), were either small-scale (Chastin et al., 2014;Dogra et al., 2016;Leask et al., 2016;McEwan et al., 2016) or interviewed older adults following active participation in a sedentary behavior intervention (Greenwood-Hickman et al., 2015), and have been largely descriptive in their approach to analysis (Chastin et al., 2014;Leask et al., 2016;McEwan et al., 2016).

To maximize the potential of interventions to effectively reduce sedentary behavior, it is essential to widen understanding of sitting and non-sitting activities within older adults’ everyday lives by including the views of a larger and more diverse sample, including older men and women of varying ages from different socioeconomic status (SES) backgrounds and with different levels of sedentary behavior. It is also important to go beyond descriptive analysis and use a conceptual theoretical approach to identify generalizable factors that appear to be amenable to change, and ways in which change might be achieved that can be tested in future intervention development research (Wight, Wimbush, Jepson, & Doi, 2016).

This study therefore aims to compare the types and context of sitting (and non-sitting) activities and their role in the daily lives of older adults who sit more than average and those who sit less. Access to two existing large study cohorts (the Lothian Birth Cohort 1936 and Twenty-07 Study; seeBenzeval et al., 2009;Deary, Gow, Pattie, & Starr, 2012 for full details of the cohorts) allowed us to interview and contrast the accounts of a large, diverse sample of community-living older adults according to gender, age, and SES, as well as their current objectively measured level of sedentary behavior.

## Research Design and Methods

This qualitative study was conducted as part of a larger interdisciplinary project using a range of objective and self-report methods to examine sedentary patterns in older adults (Seniors USP [Understanding Sedentary Patterns], http://www.gcu.ac.uk/seniorsusp). Participants in the Seniors USP project (*N* = 773) were recruited from existing Scottish study cohorts: the Lothian Birth Cohort 1936 (LBC1936:*N* = 304, aged 79 years [Late-70s]) and the West of Scotland Twenty-07 Study, which comprises three age cohorts, the two oldest of which (described as the 1950s and 1930s cohorts) were included in the Seniors USP project (1950s:*N* = 340, aged around 64 years [Mid-60s]; 1930s:*N* = 129 aged around 83 years [Mid-80s]).

**Figure 1.**
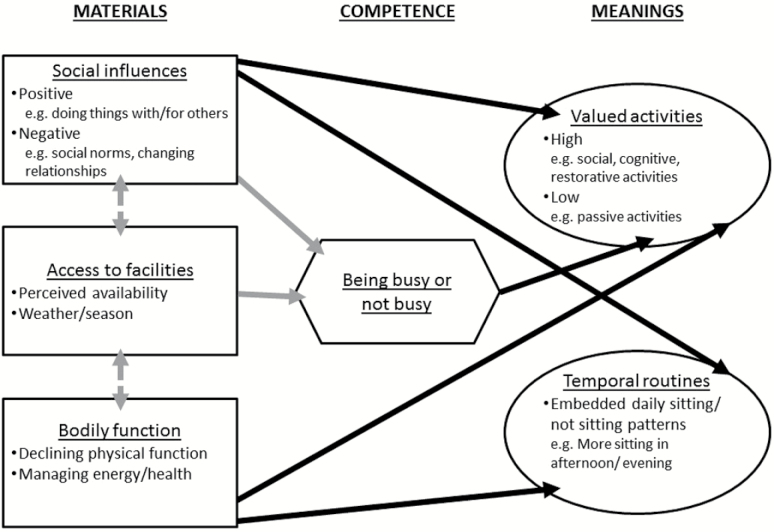
Social practice model of sedentary behavior in older adults.

In this article, we report the findings from face-to-face, semistructured interviews with a purposive subsample of 44 older adults who, as part of the Seniors USP Project, had undergone objective measurement of their sedentary behavior by wearing an activPAL accelerometer for 7 days within the previous 3 months. They had also received visual feedback on their sedentary behavior (seeSupplementary Figure 1) along with advice on how to become less sedentary (seeSupplementary Figure 2) prior to taking part in their interview. The study was approved by the University of Glasgow College of Social Sciences Ethics Committee (Ref: 400130247—for Twenty-07 participants) and the NHS Scotland A Research Ethics Committee (Ref: 07/MRE00/58—for Lothian Birth Cohort participants). All participants provided written consent to participate in the research.

### Participants and Setting

We sought to include equal numbers of men and women from the three age cohorts (Mid-60s, Late-70s, Mid-80s), of high and low SES, and with higher and lower levels of objectively measured sedentary behavior (SB) to ensure we reached a diverse sample of participants. We used a sampling frame to identify participants aiming to recruit two participants within each category (based on cohort, gender, SES, and SB), the first participants who met the criteria were invited to take part in the interview. The final numbers achieved are shown inTable 1. SES was obtained from pre-existing cohort data on previous occupation (or current occupation, if still working). The six occupational classes were as follows: (I) Professional, (II) Intermediate occupations, (III) (N) Skilled occupations (non-manual), (III) (M) Skilled occupations (manual), (IV) Partly skilled occupations, and (V) Unskilled occupations (Office of Population Censuses and Surveys, 1980). High SES participants were drawn from classes I and II, and Low SES participants were drawn from classes IV and V and III (M) (III (M) participants were included for the late-70s and mid-80s cohort due to the low numbers of low SES participants who agreed to be interviewed in these cohorts).

**Table 1.** Detailed Sample Characteristics Indicating the Number of Participants From Each Gender, Age Cohort, and SES and SB Category

	1950s (Mid-60s)				LBC1936 (Late-70s)						1930s (Mid-80s)			
	High SES		Low SES		High SES		Low SES			Other SES^a^	High SES		Low SES	
	Higher SB	Lower SB	Higher SB	Lower SB	Higher SB	Lower SB	Higher SB	Lower SB	Other SB^a^	Lower SB	Higher SB	Lower SB	Higher SB	Lower SB
Men	2	2	3	2	2	2	2	0	2	0	2	2	1	1
Women	2	2	2	1	2	2	1	2	0	1	1	2	2	1

*Note:* SB = sedentary behavior; SES = socioeconomic status.

^a^Other—participants who did not meet SB or SES criteria were included in the main analysis, but not in any between-group comparisons.

Mean objectively measured sedentary time (including sleep time) over a 24-hr period was calculated from the activPAL data obtained from the first participants from each age cohort in the main Seniors USP project. Tertiles (rather than a median split) were used to determine the lower (Lower SB) and higher (Higher SB) sedentary behavior threshold cutoff points for each cohort separately (Table 2). This procedure allowed us to maximize the difference in sedentary behavior between Higher SB and Lower SB groups. To accommodate lack of availability of Lower SB, Low SES men in the Late-70s age group, two men in this group in the middle tertile (i.e., between the higher and lower sedentary behavior cutoffs [Other SB]) were interviewed (seeTable 1). In addition, one woman in the Late-70s age group with occupational class III (N) (Other SES) was interviewed in the early stages of the study. These participants were included in the main analyses, but excluded from the between-group analysis.

**Table 2.** Mean Objectively Measured 24-Hr Sedentary Time, and Higher and Lower SB Thresholds for Each Age Cohort

	1950s	LBC1936	1930s
	*N* = 51	*N* = 92	*N* = 50
Mean sedentary time (hr)	17.2	17.7	18.3
Higher SB threshold (hr)	≥18.1	≥18.5	≥19.1
Lower SB threshold (hr)	≤16.3	≤17.3	≤17.9

*Note:* SB = sedentary behavior.

The interviews were conducted between May 2015 and June 2016 by V.J.P., who has a background in sociology and is an experienced qualitative researcher. Interviews were conducted in participants’ homes (*n* = 39) or at a clinical research facility (*n* = 5), as chosen by the participant, and lasted between 16 min and 1 hr 55 min, most interviews lasted between 30 and 40 min (mean interview length = 41 min).

### Data Collection

The interview schedule was developed to guide discussion on participants’ daily sitting and nonsitting activities, their perceptions of these activities, what was good and less good about sitting, and their views on reducing their sitting time. The visual 24-hr activPAL feedback participants had received on their daily sitting and non-sitting time during the Seniors USP Project (seeSupplementary Figure 1) was used to prompt discussion around what they did while sitting and not sitting. The interview schedule and visual feedback were piloted with two participants with no major revisions, and the pilot data were included in analysis. Fieldnotes were written up electronically immediately after each interview to capture contextual information including whether spouses/partners were present. Any (unsolicited) contributions made by spouses/partners during the interview were not included in the data analysis.

### Data Analysis

Interviews were digitally recorded and transcribed verbatim with participants’ consent. Anonymized transcripts were analyzed using a thematic framework approach (Ritchie, Spencer, & O’Connor, 2003) and NVivo10 software to organize and synthesize the data and undertake between-group comparisons according to participants’ level of sedentary behavior, gender, age, and SES (for a detailed description of the framework analysis process, seeSupplementary Table 1). A sample of transcripts (*n* = 3) was read closely by other members of the research team with expertise in qualitative methods (C.M.G., S.W.) and sedentary behavior (C.F.F., N.M.). An initial coding frame with eight broad themes was constructed, guided by the research questions and emergent ideas. The eight broad themes were as follows: Description of sitting/non-sitting activities; Perceptions of sitting/non-sitting; Health and well-being; Function; Other people; Hobbies and interests; Changing/not changing sitting behavior; and Changes in sitting over time. The coding frame was applied by V.J.P. to all transcripts, with six (14%) transcripts double coded by C.F.F. and C.M.G. Each broad theme was then analyzed by the research team and subthemes identified, which were applied by V.J.P. and charted in a series of framework matrices (Ritchie et al., 2003). The final stage of the analysis involved mapping the matrices to facilitate theoretical interpretation and between-group comparisons (age, gender, SES, and SB).

This article presents analysis of two of the eight broad themes: Description of sitting/non-sitting activities, which included older people’s accounts of the activities they did when they were sitting and not sitting, and Perceptions of sitting/non-sitting, which included what older people said about the things they did when they were sitting and not sitting. These broad themes contain information about the context, types, and understandings of older adults’ sitting that are necessary for intervention development (Wight et al., 2016). These themes were analyzed using two theoretical perspectives; the ecological models of sedentary behavior and physical activity, and social practice theory. Drawing on the ecological models of sedentary behavior (Owen et al., 2011) and physical activity (Sallis, Owen, & Fisher, 2015), which identify four domains in which sitting/non-sitting behaviors can occur, we characterized activities reported in the “Description of sitting/non-sitting activities” theme as: leisure-time; household; transport; and occupation activities (Table 3).

**Table 3.** Definition of sitting and non-sitting activities in the four domains of the ecological model of sedentary behavior

Domain	Definition
Leisure-time	Any recreational sitting or non-sitting activity
Household	Any sitting or non-sitting domestic activity associated with the day-to-day running and upkeep of the home
Transport	Any sitting or non-sitting activity associated with transportation
Occupation	Any sitting or non-sitting activity associated with work or voluntary work, including formal and informal caring responsibilities

The ecological model provides insight into the types and context of activities, which might be changed to reduce sedentary behavior, but has been criticized for failing to account for the multifactorial influences on behavior (Chastin et al., 2016). Therefore, to understand the role of sitting and non-sitting activities in older adults’ daily lives, we drew on social practice theory, which focuses on the action being performed, rather than on the individual performing it (Blue, Shove, Carmona, & Kelly, 2016). Social practice theory suggests that what people do depends on the interaction and integration of three key elements: materials (e.g., social support, facilities, a person’s physical body); competence (e.g., people’s understandings of the situation they find themselves in); and meanings (including embodied and symbolic understandings of the social significance of the practice, and past experiences;Blue et al., 2016;Shove, Watson, & Pantzar, 2012). These elements are in turn shaped by cultural expectations of appropriate practices which vary across age, gender, and SES. The social practice perspective allowed us to identify six analytical themes in the “Perceptions of sitting/not sitting” broad theme. These were, in relation to materials: “Social influences”; “Access to facilities”; and “Bodily function”; in relation to competence: “Being busy or not busy”; and in relation to meanings: “Valued activities” and “Temporal routines.”

Extracts chosen to illustrate the analyses are labeled to indicate gender (M, F), interview ID (1–44), age cohort (Mid-60s, Late-70s, Mid-80s), socioeconomic status (High SES, Low SES, Other SES), and level of sedentary behavior (Higher SB, Lower SB, Other SB).

## Results

### What Do Older Adults Do When They Are Sitting and Not Sitting?

Table 4 shows that participants described many different kinds of leisure-time, household, transport, and occupational (including voluntary work) sitting and non-sitting activities. There were no clear differences in the types of activities reported according to level of sedentary behavior; participants in the Higher SB group simply reported doing sitting activities more often or for longer than those in the Lower SB group. Most sitting activities belonged to the leisure-time domain, with many taking place at home. Watching TV was reported by all but one person, although time spent doing this ranged from less than an hour to almost all day. Other commonly reported leisure-time home-based sitting activities included reading, doing puzzles, and using computers or tablets:

**Table 4.** Older people’s sitting and non-sitting activities mapped across the four domains of the ecological model of sedentary behavior

Leisure-time domain	
*Sitting*	*Non*-*sitting*
*At home*	*Outside the home*
• Watching TV	• Walking (leisure)• Shopping• Walking dogs• Visiting attractions (e.g., museums, art galleries)• Playing sport (e.g., golf, bowls)• Structured exercise (e.g., gym, Zumba)• Going to betting shop• Going on day trips• Art/sewing classes• Going to the library• Painting• Attending community events
• Reading	
• Napping	
• Doing puzzles (e.g., Sudoku, jigsaws)	
• Using a computer/tablet (e.g., checking personal email, Facebook, browsing internet, shopping)	
• Relaxing (e.g., after activity)	
• Listening to radio/music	
• Listening to talking books	
• Knitting/sewing/crochet	
• Playing a musical instrument	
• Making models	
• Sitting in garden	
• Sitting thinking/doodling	
• Smoking	
*Outside the home*	
• Eating/drinking at cafés and restaurants	
• Going to the theatre or cinema	
• Board game clubs	
• Computer lessons	
• Learning poetry	
• Going to the pub	
• Playing bingo	
• Playing cards/board games	
• Sitting in park	
*Both sitting and non*-*sitting*	
At home	
*•*Reading the newspaper	
*•*Using a laptop	
*•*Doing crosswords	
*•*Making or receiving phone calls^a^	
Outside the home	
• Watching live sport	
Household domain	
*Sitting*	*Non*-*sitting*
• Eating meals	• Preparing meals
• Drinking tea/coffee	• Self-care (e.g., getting washed, dressed)
• Domestic administration (e.g., filing, accounts, sorting paperwork)	• Making tea/coffee
• Sorting medication	• Cleaning
• Polishing shoes	• Tidying
	• Hoovering
	• Doing dishes
	• Dusting
	• Laundry
	• Ironing
	• Making bed
	• Cleaning windows
	• Gardening
	• DIY
	• Sweeping/washing floors
	• Caring for pets (e.g., feeding, grooming)
*Both sitting and non*-*sitting*	
*•*Eating breakfast	
*•*Making phone calls^a^	
Transport domain	
*Sitting*	*Non*-*sitting*
• Bus	• Walking (all or part of a journey)
• Train	• Cycling
• Driving	
Occupation domain	
*Sitting*	*Non*-*sitting*
• Using a computer for work	• Standing/active voluntary work/job
• Driving for work	• Caring for spouse
• Administration tasks	• Caring for family members
• Preparation for voluntary work	• Helping others (e.g., friends, neighbors)

*Note:* ^a^Making phone calls was placed in both the leisure-time and household domains because some of the calls were social (e.g., to friends or family) and others were related to the day-to-day running of the home.

I’d maybe watch the telly, or play a game on my iPad, or do a crossword, or read a paper. F3, Late-70s, Other SES, Lower SB

Some leisure-time activities were reported that took place outside the home. These included some sitting activities (e.g., in cafes, restaurants, pubs, theatre, cinema), but the majority of leisure-time activities outside the home were non-sitting (e.g., shopping, visiting museums, art galleries). Playing sport (e.g., golf, bowls) and doing structured exercise (e.g., group classes) were also commonly reported, particularly by the youngest age group:

Well, I have yoga on Tuesday, and then, Wednesday, I have yoga, and my friend comes at lunchtime, and she’s here ‘till about ten o’clock. And then, Thursday, I’ve got Zumba. F29, Mid-60s, High SES, Lower SB

Lower SB participants reported doing leisure-time activities (including sitting and non-sitting) outside the home more often than Higher SB participants. There were also some gender and SES differences: only women talked about knitting, sewing and playing bingo, and were more likely to do sitting activities outside the home (e.g., in cafes, at the theatre) than men, who were more likely to do non-sitting activities (e.g., sport) when out. High SES participants reported more sitting and non-sitting leisure-time activities outside the home than Low SES participants.

In the household domain, all participants, regardless of level of SB, described (non-sitting) housework chores, and many reported integrating these into their daily routine by: “*just pottering around the house doing bits and pieces*”*F5, Late-70s, High SES, Lower SB*. Some also said that pottering encouraged them to move around the house (e.g., going up and down stairs), and helped them break up sitting time:

I don’t consciously do things, but I find it difficult to watch a film, because it’s on too long and I get bored. So I tend to watch, maybe, hour long programmes. So I’ll maybe watch a programme, and even in the middle of that, if I think I’ve got to go and do something—oh, I haven’t put that ironing away, or I haven’t made that telephone call—I’ll pause it. F18, Mid-60s, High SES, Lower SB

Some leisure-time and household activities reported as being done sitting by most participants, were described as non-sitting activities by a minority. For example, whereas most participants sat to eat breakfast, talk on the phone, read the newspaper, and do crosswords, a few said they tended to do these activities standing up. For some these were part of longstanding routines, whereas others had practical reasons for doing so (“*I do find it easier reading small print when I’m standing up.*”*F4, Late-70s, High SES, Lower SB*).

In the transport domain, both Higher and Lower SB participants reported active (mostly walking all/part of the way) and less active (by bus, train, car) travel. Lower SB participants reported more sitting and non-sitting activities than Higher SB participants in the occupation domain.

### Why Do Older Adults Spend Time Sitting and Not Sitting?

Although the ecological model is useful to classify the types of sitting and non-sitting activities reported by older adults, social practice theory can provide more insight into the multifactorial influences on these activities, and therefore help to identify sitting activities that may be amenable to change. The following sections summarize older adults’ accounts of their sitting and non-sitting activities in relation to the three key elements of social practice theory: materials, competence, and meanings (Shove et al., 2012).

## Materials

### Social Influences

The social practice analysis revealed the importance of other people in shaping when participants sat and did not sit. Lower SB participants tended to talk more about going out and doing things with other people than those in the Higher SB group. Indeed, regardless of whether the social activity itself was sedentary, doing things with other people appeared to be associated with less sitting. For example, one Lower SB woman described how simply getting ready to go out to meet friends involved many non-sitting activities:

I’d have been making breakfast, up and down, washing your hair, having your shower, that kind of thing. […] it takes me two hours to get out. F33, Mid-80s, High SES, Lower SB

The types of activities done with other people tended to differ by gender: while women often reported sitting with friends, men reported more non-sitting social activities (e.g., playing golf). Participants (often Lower SB) also described helping family members, friends, and/or neighbors:

I do have a lot of people I see […] you know, I have a friend who’s just had her hip done, so you do things for them. She stays at the other side of the city so I have to get there. I also have two elderly neighbours. I know I am not in the first flush of youth but they are about five years older than me. So, you know, I make sure they’re alright. F4, Late-70s, High SES, Lower SB

As in this woman’s account, sometimes these social support activities were informal; however, some participants reported having more formal caring roles, such as looking after grandchildren. Others, mostly High SES, described how formal voluntary work kept them busy both mentally and physically:

I’ve been doing a fair bit of things, some of which I continue to do, the main one being giving lectures on the history of [my home city] for the City Council Adult Education Programme, so that takes up a lot of time from September through to March, really. […] I hope it keeps my mind active and I hope it also keeps me active in that I’ve got to go round and get pictures for slides for the talks and things like that. M12, Late-70s, High SES, Lower SB

Although the interviews suggested that other people had a positive influence on older adults’ sedentary behavior, this was not always the case. Some participants (both Higher and Lower SB) said they felt constrained by social norms to do certain activities sitting down:

Everybody sits…I mean, if you go to a café, you can hardly stand and have a cup of tea in a café. Or if I go to the church meetings, imagine in the church if I stood, you know, during the sermon. F10, Late-70s, High SES, Higher SB

Others described how the (deteriorating) health and death of partners and friends, as well as friends moving away from the area, had reduced their opportunities for being socially active. A few Higher SB participants complained of feeling socially isolated, which one woman blamed directly for her sitting too much:

I lived here 14 years and I can honestly say, I’ve no friends, I’ve nowhere to go …So I’ve nothing to do so I just sit about. F23, Mid-60s, Low SES, Higher SB

### Access to Facilities

As this woman’s account illustrates, the perceived availability and accessibility of leisure facilities also influenced older adults’ sitting and non-sitting activities. Lower SB and High SES participants reported using leisure facilities more than Higher SB and Low SES participants, respectively. High SES participants seemed happy to travel to access facilities in other areas, whereas some Low SES participants were reluctant to leave their immediate neighborhood and tended to spend a lot of their time (sitting) at home:

There’s nothing here. It’s a nightmare. […] So if you don’t know [the area] at all, or if you do, you’ll know that it’s very limited here. Anything, you’ve got to drive to, even the swimming. You’ve got to drive to [nearby town] or that to get to the swimming or the gym or whatever, and you think, God, if it was only nearer you could maybe walk part of the way or whatever but, no, there’s not a great amount of things locally that you could go to. F25, Mid-60s, Low SES, Higher SB

Poor weather, compounded by short day length in winter, was another important factor that prevented many participants (both Higher and Lower SB) from doing leisure-time activities outside the home, therefore increasing the time they would spend at home where leisure-time activities are mostly sitting.

If the weather is bad, there is no point in going anywhere, you might as well be in the comfort of your own home. M32, Mid-60s, Low SES, Higher SB

### Bodily Function

Almost all participants (regardless of level of SB) recognized that their physical capacity influenced their sitting and non-sitting activities. Some said they felt lucky to be able to be active, but were also aware that declining physical functioning, which they often attributed to getting older, meant they were no longer able to do as much as they used to. Some overcame this limitation by using sitting to break up non-sitting activities:

Mind you that’s the way it’s turning out now, because you get older. You used to be able to do [all the housework] in a oner, whereas now you have to have wee seats in between. F17, Mid-60s, High SES, Higher SB

Participants also described using sitting to help them manage (sometimes multiple) health issues:

My eyesight is awful and I’m on the blind register now and I have trouble with my back, and it means that if I do too much I get a lot of pain. So I can therefore only walk for about half an hour and I have to sit down. F39, Mid-80s, High SES, Higher SB

However, interviewees (in both SB groups) were aware of the negative impact of sitting on their bodies; many described how sitting for a long time resulted in stiffness, discomfort, or even pain. For some this was an indication that they had sat too long:

I think when you sit a long time you get stiff. I think it’s much better to be active and move about because if you’ve got an ache or a pain and you walk about, it goes away whereas the longer you sit, the stiffer you get so, I think it is much better to be active. F14, Late-70s, Low SES, Lower SB

### Competence

#### Being Busy or Not Busy

Participants from both Higher and Lower SB groups made the distinction between being busy and not busy rather than between sitting and not sitting when making sense of what they did during the day. Therefore, sitting activities were often described as “*something to do*”*M8, Late-70s, Low SES, Higher SB*, and participants were keen to emphasize that they were busy even though they were sitting down:

I mean, I’m not sitting staring into space, so I’m doing something, you know. I’m active, mentally, as well. So I don’t sit there being bored. M27, Mid-60s, High SES, Lower SB

The distinction between being busy or not busy may be related in part to older adults’ perceptions of their cognitive functioning and mental well-being. Some participants (particularly the Lower SB group) described how important it was to them that they had things to do:

Well you have to keep active. I would go spare if I had to be… No. I worked as a living, that I was out every day and I had an elderly parent I looked after at the same time, yeah, but I’ve never…I wouldn’t really like to be…I would not have liked to have to be staying in all the time, no, no, I couldn’t do that, no, no. F4, Late-70s, High SES, Lower SB

Some Higher SB participants had personal accounts of how sitting doing nothing for long periods of time led to them feeling low or depressed. Those from the youngest age group, in particular, talked about the negative impact of sitting on their mental health:

I think the longer you sit the lower you get. If I’m on the move then I do feel better, but if I’ve been sitting in a chair you do get bored. M31, Mid-60s, High SES, Higher SB

### Meanings

#### Value of Activities

Being busy and being seen to be busy was therefore extremely important to the older adults interviewed. Participants, regardless of level of SB, also talked about the value, or lack of value, they associated with different activities. Sitting activities associated with social, cognitive, and/or restorative benefits (e.g., going for coffee with friends, doing crosswords, sitting to relax) were highly valued. Most described how they derived some of these benefits from watching TV, as this woman’s account illustrates:

I would say between 8:00 and 9:00 is the point at which we say, right, are you free, am I free, right, let’s watch some of the programmes I’ve got recorded […] it’s how we [my husband and I] relax I suppose in the evenings. F22, Mid-60s, High SES, Higher SB

However, passive TV viewing (i.e., not watching anything particular) was considered a low-value activity:

I: So on this day then you’ve got, kind of, from two o’clock there onwards this is a lot more sitting. So what sort of things are you doing then?

Watching telly. Definitely watching television and that’s the bane of my life. I should really put it out, because your time passes so quickly when you watch that, and it’s wrong. F17, Mid-60s, High SES, Higher SB

Other passive sitting activities were also seen as low value. These tended to be less purposeful (i.e., not busy) activities and included some types of napping (e.g., dozing), sitting thinking or doodling, and some types of computer use (e.g., browsing the internet without a specific purpose).

#### Temporal Routines

Finally, participants across SB groups described several factors that contributed to the formation of deeply embedded daily sitting/non-sitting routines. Many reported how socially shaped routines (e.g., social norms around mealtimes) resulted in the formation of temporal patterns of sitting that did not vary much from day-to-day:

I have my dinner about…approximately six…before six. And I have my dinner. And I’ll maybe sit and I’ll watch the news at six o’clock. And I’ll say, I’ll get these dishes done before my…you know how your soaps usually start about half past seven…then watch [TV at] half past seven. F41, Mid-80s, High SES, Lower SB

Most participants also described sitting more in the afternoon and evening, than in the morning. For many, these temporal routines had emerged as a way of managing their (declining) physical function and rewarding themselves for doing most of their (often household) non-sitting activities earlier in the day:

About 11am, having a coffee probably, especially if I’ve been shopping, I come back and sometimes sit just to get my breath back and get my strength back, that’s why I sit around that time, because I’ve usually been doing things, even if it’s only going up to the shops or, as I say, hoovering. But, yes, I do sit about 11 o’clock, yes. F40, Mid-80s, Low SES, Higher SB

## Discussion

This qualitative study has found that the types of sitting (and non-sitting) activities done by older people with higher and lower levels of sedentary behavior do not differ. Those with lower levels of sedentary behavior simply described doing fewer sitting activities than those with higher levels, across the ecological model leisure-time, household, transport, and occupational domains (Owen et al., 2011). Most sitting activities were leisure-time activities; many of these took place in the home, but “pottering” around doing nonsitting household chores helped some older adults break up long periods of sitting.

Social practice theory allowed us to gain a deeper understanding of the multiple, and often highly inter-relational, factors that influence older adults’ sitting and non-sitting activities throughout the day. Older adults with lower levels of sedentary behavior reported doing more things with/for other people than those with higher levels of sedentary behavior. Social activities often took place outside the home, but some low SES participants reported a lack of things to do locally (or people to do them with) led them to sit more. Participants’ accounts highlighted the importance of being busy (and being seen to be busy), regardless of whether they were sitting or not; however, declining physical function and health placed some restrictions on their ability to be active (and therefore busy) both within and outside the home.

Our social practice model of sedentary behavior (seeFigure 1) summarizes how materials and competence influence each other, and inform the symbolic meanings that older adults associate with sitting activities. For example, being busy (including with other people) was associated with cognitive and social benefits derived from high-value sitting activities, whereas less busy (passive) sitting activities were not valued by participants. Declining bodily function was associated with the restorative benefits of sitting, which contributed to the formation of embedded daily temporal routines, often involving older adults sitting more in the afternoon or evening to recover from, or relax after, non-sitting activities earlier in the day.

Our study adds to understandings of the type, context, and role of sitting and non-sitting activities in older adults’ daily lives, and how these might be used to inform interventions to reduce sedentary behavior in a number of ways. First, although the types of sitting and non-sitting activities reported were consistent with previous research (Leask et al., 2015;Lenz, 2014), our study highlighted that some typically sitting activities (e.g., reading the newspaper, doing crosswords, making and receiving phone calls, eating breakfast) were done by some people standing up. Such examples could be used to help older adults identify ways they could incorporate more non-sitting activities in their daily lives.

Second, although it is commonly reported that social pressure from other people (family, friends, carers) results in older adults sitting more (Chastin et al., 2014;Greenwood-Hickman et al., 2015), our social practice analysis suggested that socializing with other people may help older adults to sit less. It is therefore important that people are supported to remain socially active as they grow older to reduce the time they sit (often alone) at home. Supporting older adults to remain socially active will not only support them to sit less, but will also reduce social isolation, which is associated with poor health (Hawkley & Cacioppo, 2010).

Third, previous research has shown that older adults value some sitting activities (e.g., seen as worthwhile or enjoyable) more than others (Chastin et al., 2014;Dogra et al., 2016;Greenwood-Hickman et al., 2015;McEwan et al., 2016). Our study reveals the importance of the distinction between being busy and not busy in underpinning these judgments. Doing things with other people (social) and the cognitive benefits of busy (sitting and non-sitting) activities, as well as the restorative function of sitting, were all particularly valued by older adults. “Not busy” (less purposeful) sitting activities were viewed as low value, often simply as a way of filling time, thus supporting the conclusions of a recent systematic review of sedentary behavior that interventions for older adults should target passive sitting (Wullems et al., 2016).

Previous evidence from time-lapse cameras suggests that many older adults tend sit more in the afternoon and evening (Leask et al., 2015), and our study reveals that declining physical function (as well as socially shaped routines) contribute to these temporal patterns of sitting and non-sitting activities. Almost all participants talked about how reduced physical functioning (compared with when they were younger) meant they were sitting more as they grew older, but similar to previous qualitative studies, they also described the negative impact of sitting too long on their physical health (Chastin et al., 2014;Dogra et al., 2016;Leask et al., 2016;McEwan et al., 2016). One way of reducing home-based sedentary behavior may therefore be to encourage older adults to think about how they can reduce stiffness and discomfort by breaking up long periods of passive sitting in the afternoon or evening by spreading non-sitting household chores throughout the day.

Finally, although access to facilities has previously emerged as a barrier to older people reducing their sedentary behavior (Chastin et al., 2014;Dogra et al., 2016), it was clear from our study that perceived inaccessibility was more of a problem for low SES participants. This novel finding suggests that sedentary behavior interventions targeting older people from low SES areas should have a particular focus on changing perceptions about accessing facilities (e.g., providing detailed, personalized, information about local resources, and free bus travel to access facilities elsewhere), and encouraging their use (e.g., by supporting them to go along with other people). One approach might be through age-friendly community initiatives (Kendig & Phillipson, 2014) to support older people to engage with facilities in their area. Although some participants (particularly men) in our study reported playing sport, analysis of Scottish Health Survey data suggest that these are in a minority (Strain et al., 2016). Therefore, other leisure activities (e.g., based around walking, hobbies, cultural visits) may have more appeal to encourage some older people to get out of the house, particularly if support can be provided to encourage them to attend.

This study had several strengths: being part of the Seniors USP project allowed us to recruit a large sample of men and women from different age groups and SES backgrounds, and to sample participants according to their current (objectively measured) level of sedentary behavior. Our conceptual theoretical approach allowed us to identify generalizable factors that are amenable to change and ways of incorporating them in future sedentary behavior interventions for older adults.

There were some limitations: first, use of previously collected cohort data meant SES based on (often previous) employment status was not always a true representation of older people’s current socioeconomic circumstances. Second, data used to determine the higher and lower sedentary behavior threshold cutoff points were obtained over 24 hr and included night-time sleeping, which is not classed as a sedentary behavior. However, the use of tertile cutoff points (rather than a median split) maximized the difference between Higher SB and Lower SB groups, and thus minimized any misclassification. Finally, all participants had received advice on how to become less sedentary (seeSupplementary Figure 2) along with the visual feedback on their sedentary behavior (seeSupplementary Figure 1) before taking part in the interviews. They were therefore aware of guidance to become less sedentary, which may have influenced both the types of sitting and non-sitting activities they reported doing, and how they reported their perceptions of sedentary behavior (i.e., these may have appeared more negative than if they had been made aware of the sedentary behavior guidance).

In conclusion, as many sedentary activities are embedded in older adults’ lives as part of their daily temporal routines and/or associated with cognitive, social, or restorative benefits, they may be difficult to change. Our study suggests that promising strategies may be to reduce passive sitting by first, supporting older people to go out more by developing new social connections, and engaging with local facilities and other resources (e.g., community groups). Second, by encouraging them to think about ways to break up long periods of passive sitting with non-sitting activities (e.g., doing some sitting activities standing up, spreading household chores throughout the day).

## Funding

This work was supported by the UK Medical Research Council (MRC) as part of the Lifelong Health and Wellbeing Initiative (LLHW; MR/K025023/1). LBC1936 data collection was supported by Age UK (G1001245/96077) and MRC (Mr/M01311/1), undertaken within the University of Edinburgh Centre for Cognitive Ageing and Cognitive Epidemiology supported by the Biotechnology and Biological Sciences Research Council and MRC as part of the LLHW (MR/K026992/1). The West of Scotland Twenty-07 data collection was supported by the MRC and undertaken by the MRC Social and Public Health Sciences Unit (MC_A540_53462).

## Conflict of Interest

None reported.

## Supplementary Material

gny020_suppl_supplementary-material
