# Supplementary material for: What Do Older People Do When Sitting and Why? Implications for Decreasing Sedentary Behavior
Source: Gerontologist. 2018 May 15;59(4):686–97. doi: 10.1093/geront/gny020 (PMC6630262; doi:10.1093/geront/gny020)
Supplement: gny020_suppl_supplementary-material [file gny020_suppl_supplementary-material.docx]

# Supplementary Material

**Supplementary Figure 1.** Visual 24-hour activPAL^TM^ feedback participants received on their daily sitting and non-sitting time


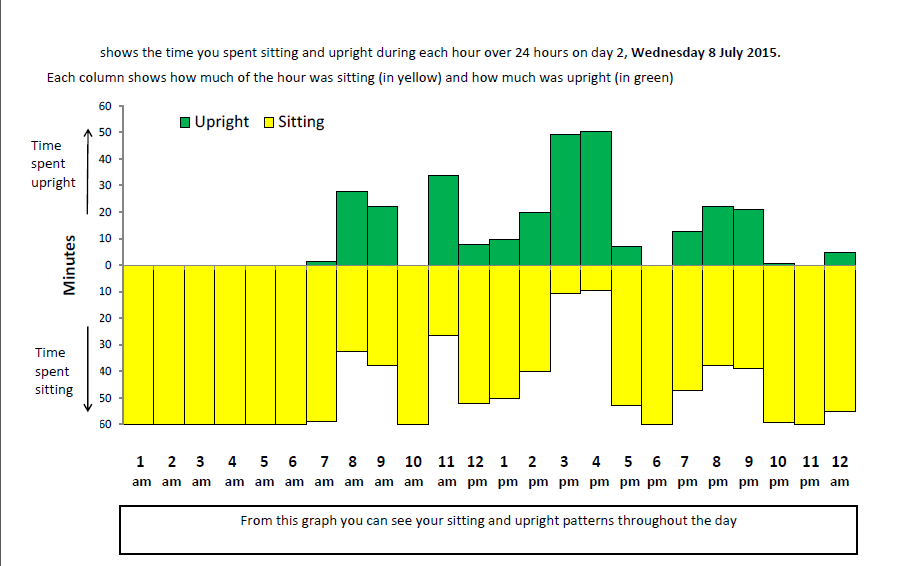


**Supplementary Figure 2.** Advice on how to become less sedentary participants received as part of Seniors USP study

**Older adults are advised to reduce their Sedentary Behaviour and avoid very long periods of sitting^1^**


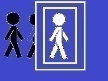

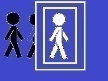

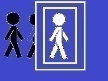

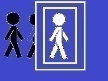

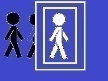

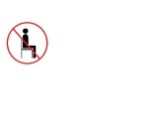

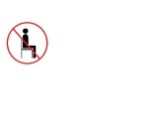

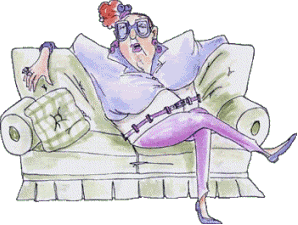

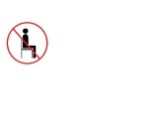

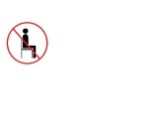

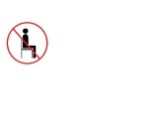

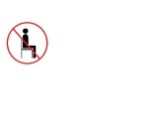


**Top Tips to break prolonged sitting**

**Just before you sit down in the evening, ask yourself a few simple questions:**

- Have I been more active today than yesterday?
- Have I sat a lot today already?
- Is there something else I could do before I sit down?
- When will I next get up again?

**Other ways of being more physically active:**

Park further away from the entrance to a shopping centre

Stand up on the bus/train for one stop or more

Walk to visit a neighbour instead of phoning them

Stand in the kitchen while the kettle is boiling

Try to get out of the house at least twice a day for a short

walk around your neighbourhood

**Some ways to reduce long periods of sitting & reduce total sitting time in a day:**

- Stand up during advertisement breaks while watching TV
- Put the remote control next to the TV rather than next to you
- Stand up after finishing a chapter of your book or a section of the newspaper
- Set an alarm to remind you to move regularly if you are on the computer
- Stand up and move around while talking on the phone
- Stand up to read the mail


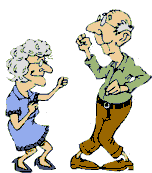
**Supplementary Table 1:** Detailed description of data analysis using thematic framework approach.

**Treat the seat**

**as a treat**

**1.** CMO (2011) Start Active, Stay Active. DoH.

Over time, by minimising the amount of time you spend sitting, standing up more during long periods of sitting (so strengthening your leg muscles), and doing a bit more activity each day, you will start to feel the **benefits to both body and mind**.

**Feel the benefit**

**of getting up**

**more often**

| **Stages of Thematic Framework Analysis^a^** | **Description of data analysis process** |
| --- | --- |
| **Familiarisation**  Immersion in data to identify key themes | - VJP listened to all interviews and checked transcripts for accuracy - VJP conducted in-depth reading of all transcripts and field notes to identify key themes and ideas - CMG, CF, NM and SW conducted and in-depth reading of a sub-sample of transcripts (N=3) to identify key themes and ideas |
| **Identifying a thematic framework**  Identifying all the key issues, definitions and themes by which the data can be examined and referenced | - The research team met to discuss and agree key themes that had emerged from the familiarisation process - Eight broad themes were identified guided by the research questions and emergent ideas from the data - Each theme was then further analysed to identify sub-themes - VJP analysed each theme identifying sub-themes - CMG, CF, NM and SW each received a sample of data from each theme to identify sub-themes - The research team met to discuss and refine sub-themes |
| **Indexing**  Applying the thematic framework systematically to all the data | - VJP applied thematic framework to the data using Nvivo 10 to code data - 6 (14%) transcripts were double coded by CMG and CF to check for accuracy |
| **Charting**  Rearranging the data according to the framework and forming charts containing distilled summaries of the data | - A series of framework matrices were created for each of the broad codes using Nvivo 10 software - VJP entered summaries of the data in to the matrices - Matrices were shared with the research team |
| **Mapping and interpretation**  Using charts to define concepts, map the range and nature of the phenomena, create typologies and interpret findings | - Nvivo 10 was used to sort matrices to allow for between-group comparison by age, gender, SES and SB - Two theoretical perspectives used to assist in interpretation of data and to identify analytical categories/themes in the data |

^a^ Stages of thematic framework analysis descriptions adapted from Pope, Ziebland & Mays, (2000).

**References:** Pope, C., Ziebland, S., & Mays, N. (2000). Analysing qualitative data. BMJ : British Medical Journal, 320(7227), 114–116;
